# Crystal structure of (*Z*)-3-(4-meth­oxy­benzyl­idene)-2,3-di­hydro­benzo[*b*][1,4]thia­zepin-4(5*H*)-one

**DOI:** 10.1107/S2056989014026267

**Published:** 2015-01-01

**Authors:** V. Vinayagam, J. Mohan Raj, S. Murugavel, R. Selvakumar, M. Bakthadoss

**Affiliations:** a Department of Organic Chemistry, University of Madras, Maraimalai Campus, Chennai 600 025, India; b Department of Physics, C. Abdul Hakeem College of Engineering & Technology, Melvisharam, Vellore 632 509, India; c Department of Physics, Thanthai Periyar Government Institute of Technology, Vellore 632 002, India; d Department of Chemistry, Pondicherry University, Puducherry 605 014, India

**Keywords:** crystal structure, benzo[*b*][1,4]thia­zepin-4(5*H*)-one, pharmaceutical properties, thia­zepin derivatives, hydrogen bonding

## Abstract

In the title compound, C_17_H_15_NO_2_S, the two C atoms linking the S and carbonyl C atoms of the seven-membered thia­zepine ring are disordered over two sites, with occupancies of 0.511 (4) and 0.489 (4); both disorder components adopt distorted twist-boat conformations. In the crystal, N—H⋯O and C—H⋯O hydrogen bonds link inverted-related mol­ecules into dimers, incorporating *R*_1_^2^(6) and *R*_2_^2^(8) ring motifs; the acceptor carbonyl O atom is bifurcated. These dimers are further linked by C—H⋯O hydrogen bonds, forming supra­molecular tapes running along the *a* axis.

## Related literature   

For the pharmaceutical properties of thia­zepin derivatives, see: Lončar-Tomascovic *et al.* (2000 ▸); Rajsner *et al.* (1971 ▸); Metys & Metysová (1965 ▸). For related structures, see: Lakshmanan *et al.* (2012 ▸); Selvakumar *et al.* (2012 ▸).
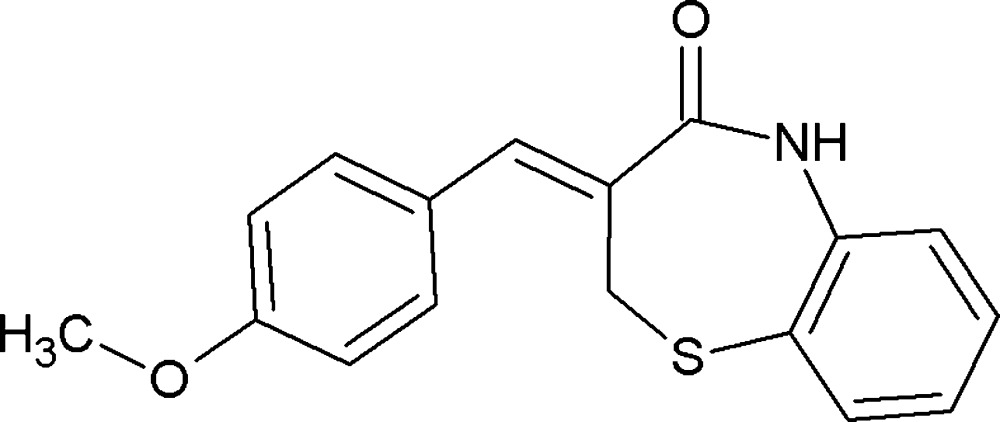


## Experimental   

### Crystal data  

C_17_H_15_NO_2_S
*M* *_r_* = 297.36
Monoclinic, 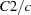

*a* = 21.434 (5) Å
*b* = 5.715 (4) Å
*c* = 23.870 (5) Å
β = 101.091 (4)°
*V* = 2869 (2) Å^3^
*Z* = 8
Mo *K*α radiation
μ = 0.23 mm^−1^
*T* = 293 K
0.30 × 0.30 × 0.25 mm

### Data collection  

Bruker APEXII CCD diffractometer
Absorption correction: multi-scan (*SADABS*; Sheldrick, 1996 ▸) *T*_min_ = 0.934, *T*_max_ = 0.944
17286 measured reflections
4099 independent reflections
2744 reflections with *I* > 2σ(*I*)
*R*_int_ = 0.028

### Refinement  

*R*[*F* ^2^ > 2σ(*F* ^2^)] = 0.045
*wR*(*F* ^2^) = 0.139
*S* = 1.03
4099 reflections
210 parameters
4 restraints
H-atom parameters constrained
Δρ_max_ = 0.25 e Å^−3^
Δρ_min_ = −0.34 e Å^−3^

### 

Data collection: *APEX2* (Bruker, 2004 ▸); cell refinement: *APEX2* and *SAINT* (Bruker, 2004 ▸); data reduction: *SAINT* and *XPREP* (Bruker, 2004 ▸); program(s) used to solve structure: *SHELXS97* (Sheldrick, 2008 ▸); program(s) used to refine structure: *SHELXL97* (Sheldrick, 2008 ▸); molecular graphics: *ORTEP-3 for Windows* (Farrugia, 2012 ▸); software used to prepare material for publication: *SHELXL97* and *PLATON* (Spek, 2009 ▸).

## Supplementary Material

Crystal structure: contains datablock(s) global, I. DOI: 10.1107/S2056989014026267/tk5350sup1.cif

Structure factors: contains datablock(s) I. DOI: 10.1107/S2056989014026267/tk5350Isup2.hkl

Supporting information file. DOI: 10.1107/S2056989014026267/tk5350Isup3.cml

**Fig. 1.**
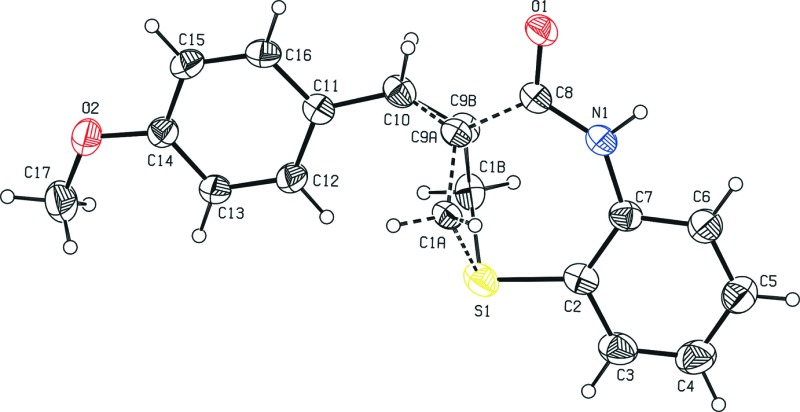
Molecular structure of the title compound showing displacement ellipsoids at the 30% probability level. H atoms are presented as a small spheres of arbitrary radii.

**Fig. 2.**
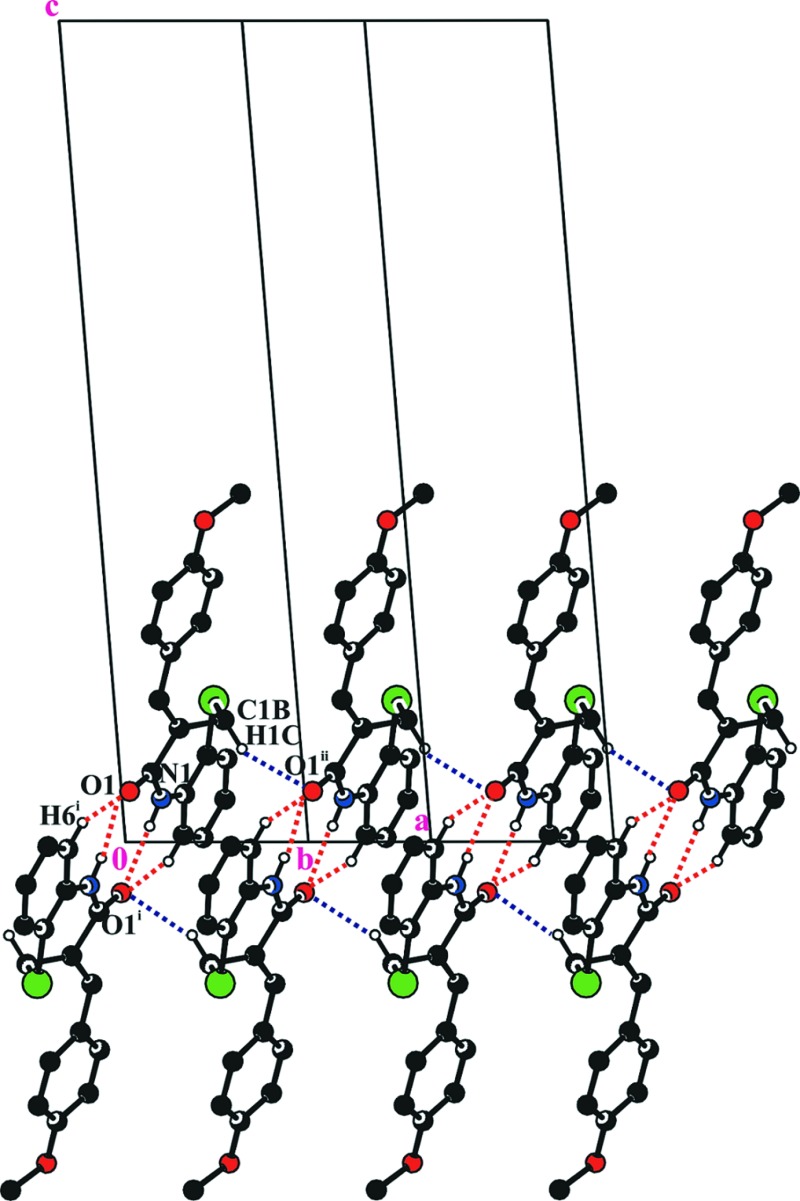
Supramolecular tape formation in the crystal packing of the title compound whereby bifurcated hydrogen bonds link inverted molecules into dimers sustained by N—H···O and C—H···O (red dashed lines) contacts are linked via C—H···O contacts (blue dashed lines) along a axis. [Symmetry code: (i) -x, -y, -z; (ii) x, 1+y, z].

. DOI: 10.1107/S2056989014026267/tk5350fig1.tif
Mol­ecular structure of the title compound showing displacement ellipsoids at the 30% probability level. H atoms are presented as a small spheres of arbitrary radii.

via a -x, −y, −z x, 1+y, z . DOI: 10.1107/S2056989014026267/tk5350fig2.tif
Supra­molecular tape formation in the crystal packing of the title compound whereby bifurcated hydrogen bonds link inverted mol­ecules into dimers sustained by N—H⋯O and C—H⋯O (red dashed lines) contacts are linked *via* C—H⋯O contacts (blue dashed lines) along *a* axis. [Symmetry code: (i) *-x, −y, −z*; (ii) *x, 1+y, z*].

CCDC reference: 1036763

Additional supporting information:  crystallographic information; 3D view; checkCIF report

## Figures and Tables

**Table 1:** Hydrogen-bond geometry (, )

*D*H*A*	*D*H	H*A*	*D* *A*	*D*H*A*
N1H1O1^i^	0.86	2.07	2.9291(18)	177
C6H6O1^i^	0.93	2.45	3.263(3)	146
C1*B*H1*C*O1^ii^	0.97	2.52	3.377(4)	147

Symmetry codes: (i) 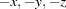; (ii) 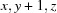.

## supplementary crystallographic information

### S1. Structural commentary

The title compound is used as an inter­mediate for the synthesis of dosulepin, which is an anti­depressant of the tricyclic family. Dosulepin prevents reabsorbing of serotonin and noradrenaline in the brain, helps to prolong the mood lightening effect of any released noradrenaline and serotonin, thus relieving depression. Dibenzo[c,e]thia­zepin derivatives exhibit chiroptical properties (Tomascovic *et al.*, 2000). Dibenzo[b,e]thia­zepin-5,5-dioxide derivatives possess anti­histaminic and anti­allergenic activities (Rajsner *et al.*, 1971). Benzene thia­zepin derivatives are identified as a type of effective anti­histaminic compounds (Metys *et al.*, 1965). In view of this biological importance, the crystal structure of the title compound has been carried out and the results are presented here.

Fig. 1 shows a displacement ellipsoid plot of (I), with the atom numbering scheme. The geometric parameters of the title molecule agree well with those reported for similar structures (Selvakumar *et al.*., 2012; Lakshmanan *et al.*, 2012). The sum of angles at N1 atom of the thia­zepin ring (359.9°) is in accordance with *sp*^2^ hybridization. Both the major and minor conformers of the disorderd thia­zepine ring adopt distorted twist-boat conformations.

In the crystal, inter­molecular bifurcated acceptor N1—H1···O1^i^ and C6—H6···O1^i^ (Table 1) hydrogen bonds link inverted-related molecules into dimers, incorporating *R_1_^2^*(6) and *R_2_^2^*(8) ring motifs. These dimers are further linked by C1B—H1C···O1^ii^ (Table 1) hydrogen bonds forming supra­molecular tapes running along the *a* axis (Fig. 2).

### S2. Synthesis and crystallization

A mixture of (*Z*)-methyl 2-(bromo­methyl)-3-(4-meth­oxy­phenyl)­acrylate (2 mmol) and *o*-amino­thio­phenol (2 mmol) in the presence of potassium *tert*-butoxide (4.8 mmol) in dry THF (10 ml) was stirred at room temperature for 1 h. After the completion of the reaction as indicated by TLC, the reaction mixture was concentrated and the resulting crude mass was diluted with water (20 ml) and extracted with ethyl acetate (3 *x* 20 ml). The organic layer was washed with brine (2 *x* 20 ml) and dried over anhydrous sodium sulfate. It was then concentrated to successfully provide the crude final product ((*Z*)-3-(4-meth­oxy­benzyl­idene)-2,3-di­hydro­benzo[*b*][1,4] thia­zepin-4(5*H*)-one). This was purified by column chromatography on silica gel with ethyl­acetate/hexane 1:19 as eluent to afford the title compound in good yield (47 %). Single crystals suitable for X-ray diffraction were obtained by slow evaporation of its ethyl­acetate solution at room temperature.

### S3. Refinement

Atoms C1 and C9 of the thia­zepine ring are disordered over two positions (C1A/C1B and C9A/C9B) with refined occupancies of 0.511 (4) and 0.489 (4). The corresponding bond distances involving the disorderd atoms were restrained to be equal. H atoms were positioned geometrically, (C—H = 0.93–0.97 Å and N—H = 0.86 Å) constrained to ride on their parent atom, with *U*_iso_(H)=1.5*U*_eq_ for methyl H atoms and 1.2*U*_eq_(C) for other H atoms.

### Crystal data

**Table:**

C_17_H_15_NO_2_S	*F*(000) = 1248
*M**_r_* = 297.36	*D*_x_ = 1.377 Mg m^−^^3^
Monoclinic, *C*2/*c*	Mo *K*α radiation, λ = 0.71073 Å
Hall symbol: -C 2yc	Cell parameters from 4132 reflections
*a* = 21.434 (5) Å	θ = 1.7–29.9°
*b* = 5.715 (4) Å	µ = 0.23 mm^−^^1^
*c* = 23.870 (5) Å	*T* = 293 K
β = 101.091 (4)°	Block, colourless
*V* = 2869 (2) Å^3^	0.30 × 0.30 × 0.25 mm
*Z* = 8	

### Data collection

**Table:**

Bruker APEXII CCD diffractometer	4099 independent reflections
Radiation source: fine-focus sealed tube	2744 reflections with *I* > 2σ(*I*)
Graphite monochromator	*R*_int_ = 0.028
Detector resolution: 10.0 pixels mm^-1^	θ_max_ = 29.9°, θ_min_ = 1.7°
ω scans	*h* = −30→24
Absorption correction: multi-scan (*SADABS*; Sheldrick, 1996)	*k* = −7→8
*T*_min_ = 0.934, *T*_max_ = 0.944	*l* = −33→32
17286 measured reflections	

### Refinement

**Table:**

Refinement on *F*^2^	Primary atom site location: structure-invariant direct methods
Least-squares matrix: full	Secondary atom site location: difference Fourier map
*R*[*F*^2^ > 2σ(*F*^2^)] = 0.045	Hydrogen site location: inferred from neighbouring sites
*wR*(*F*^2^) = 0.139	H-atom parameters constrained
*S* = 1.03	*w* = 1/[σ^2^(*F*_o_^2^) + (0.0647*P*)^2^ + 1.1615*P*] where *P* = (*F*_o_^2^ + 2*F*_c_^2^)/3
4099 reflections	(Δ/σ)_max_ < 0.001
210 parameters	Δρ_max_ = 0.25 e Å^−^^3^
4 restraints	Δρ_min_ = −0.34 e Å^−^^3^

### Special details

**Table:**

Geometry. All esds (except the esd in the dihedral angle between two l.s. planes) are estimated using the full covariance matrix. The cell esds are taken into account individually in the estimation of esds in distances, angles and torsion angles; correlations between esds in cell parameters are only used when they are defined by crystal symmetry. An approximate (isotropic) treatment of cell esds is used for estimating esds involving l.s. planes.
Refinement. Refinement of F^2^ against ALL reflections. The weighted R-factor wR and goodness of fit S are based on F^2^, conventional R-factors R are based on F, with F set to zero for negative F^2^. The threshold expression of F^2^ > 2sigma(F^2^) is used only for calculating R-factors(gt) etc. and is not relevant to the choice of reflections for refinement. R-factors based on F^2^ are statistically about twice as large as those based on F, and R- factors based on ALL data will be even larger.

### Fractional atomic coordinates and isotropic or equivalent isotropic displacement parameters (Å^2^)

**Table:**

	*x*	*y*	*z*	*U*_iso_*/*U*_eq_	Occ. (<1)
C2	−0.05513 (8)	0.5769 (3)	0.10567 (7)	0.0516 (4)	
C3	−0.09963 (11)	0.7568 (3)	0.10107 (9)	0.0689 (5)	
H3	−0.1001	0.8512	0.1327	0.083*	
C4	−0.14266 (12)	0.8004 (4)	0.05193 (10)	0.0790 (6)	
H4	−0.1718	0.9219	0.0503	0.095*	
C5	−0.14211 (10)	0.6629 (4)	0.00527 (10)	0.0722 (5)	
H5	−0.1708	0.6906	−0.0286	0.087*	
C6	−0.09941 (10)	0.4851 (4)	0.00854 (9)	0.0691 (5)	
H6	−0.0996	0.3927	−0.0236	0.083*	
C7	−0.05515 (8)	0.4357 (3)	0.05837 (8)	0.0525 (4)	
C8	0.03347 (9)	0.1194 (3)	0.08459 (7)	0.0549 (4)	
C10	0.10676 (9)	0.0681 (3)	0.17287 (7)	0.0528 (4)	
H10A	0.1284	−0.0130	0.1487	0.063*	0.489 (4)
H10B	0.1093	−0.0785	0.1566	0.063*	0.511 (4)
C11	0.14272 (8)	0.0921 (3)	0.23129 (7)	0.0474 (4)	
C12	0.13743 (8)	0.2801 (3)	0.26731 (7)	0.0534 (4)	
H12	0.1074	0.3957	0.2549	0.064*	
C13	0.17552 (8)	0.2999 (3)	0.32082 (7)	0.0525 (4)	
H13	0.1710	0.4276	0.3439	0.063*	
C14	0.22010 (8)	0.1302 (3)	0.33987 (7)	0.0492 (4)	
C15	0.22513 (8)	−0.0627 (3)	0.30585 (8)	0.0551 (4)	
H15	0.2542	−0.1806	0.3190	0.066*	
C16	0.18736 (8)	−0.0793 (3)	0.25292 (8)	0.0533 (4)	
H16	0.1915	−0.2093	0.2305	0.064*	
C17	0.26254 (13)	0.3437 (5)	0.42387 (10)	0.0895 (7)	
H17A	0.2716	0.4752	0.4017	0.134*	
H17B	0.2950	0.3305	0.4576	0.134*	
H17C	0.2220	0.3656	0.4346	0.134*	
N1	−0.01563 (7)	0.2418 (3)	0.05307 (6)	0.0610 (4)	
H1	−0.0258	0.1826	0.0194	0.073*	
O1	0.05465 (6)	−0.0461 (2)	0.06174 (5)	0.0605 (3)	
O2	0.26115 (6)	0.1372 (2)	0.39107 (6)	0.0691 (4)	
S1	−0.00374 (3)	0.55138 (8)	0.17212 (2)	0.06381 (18)	
C1A	0.00694 (16)	0.2393 (5)	0.18212 (13)	0.0438 (8)	0.489 (4)
H1A	0.0222	0.2080	0.2224	0.053*	0.489 (4)
H1B	−0.0340	0.1629	0.1710	0.053*	0.489 (4)
C9A	0.05207 (17)	0.1363 (7)	0.14902 (14)	0.0425 (8)	0.489 (4)
C1B	0.06528 (16)	0.4832 (6)	0.14859 (14)	0.0529 (9)	0.511 (4)
H1C	0.0661	0.5669	0.1134	0.063*	0.511 (4)
H1D	0.1016	0.5338	0.1768	0.063*	0.511 (4)
C9B	0.07040 (18)	0.2265 (6)	0.13852 (15)	0.0467 (8)	0.511 (4)

### Atomic displacement parameters (Å^2^)

**Table:**

	*U*^11^	*U*^22^	*U*^33^	*U*^12^	*U*^13^	*U*^23^
C2	0.0612 (10)	0.0418 (8)	0.0559 (9)	−0.0078 (7)	0.0218 (8)	−0.0015 (7)
C3	0.0945 (15)	0.0501 (10)	0.0679 (12)	0.0085 (10)	0.0301 (11)	0.0005 (9)
C4	0.0937 (16)	0.0612 (12)	0.0861 (15)	0.0218 (11)	0.0275 (13)	0.0105 (11)
C5	0.0695 (12)	0.0685 (13)	0.0761 (13)	0.0072 (10)	0.0079 (10)	0.0050 (11)
C6	0.0674 (12)	0.0676 (12)	0.0677 (12)	0.0064 (10)	0.0018 (9)	−0.0141 (10)
C7	0.0505 (9)	0.0505 (9)	0.0570 (9)	−0.0039 (7)	0.0117 (7)	−0.0085 (7)
C8	0.0606 (10)	0.0570 (10)	0.0470 (9)	0.0012 (8)	0.0105 (8)	−0.0085 (7)
C10	0.0680 (11)	0.0419 (8)	0.0494 (9)	−0.0073 (8)	0.0134 (8)	−0.0044 (7)
C11	0.0530 (9)	0.0431 (8)	0.0481 (8)	−0.0056 (7)	0.0147 (7)	−0.0008 (6)
C12	0.0564 (10)	0.0456 (9)	0.0562 (9)	0.0089 (7)	0.0060 (8)	−0.0005 (7)
C13	0.0556 (10)	0.0487 (9)	0.0530 (9)	0.0055 (7)	0.0097 (7)	−0.0071 (7)
C14	0.0464 (8)	0.0509 (9)	0.0514 (9)	0.0014 (7)	0.0122 (7)	0.0047 (7)
C15	0.0538 (10)	0.0464 (9)	0.0658 (11)	0.0106 (7)	0.0135 (8)	0.0047 (8)
C16	0.0601 (10)	0.0418 (8)	0.0614 (10)	0.0026 (7)	0.0200 (8)	−0.0054 (7)
C17	0.1079 (18)	0.0866 (16)	0.0624 (12)	0.0135 (14)	−0.0125 (12)	−0.0123 (12)
N1	0.0660 (9)	0.0634 (9)	0.0499 (8)	0.0091 (7)	0.0020 (7)	−0.0183 (7)
O1	0.0781 (8)	0.0546 (7)	0.0481 (7)	0.0079 (6)	0.0104 (6)	−0.0085 (5)
O2	0.0680 (8)	0.0720 (9)	0.0597 (8)	0.0116 (7)	−0.0064 (6)	0.0002 (7)
S1	0.0906 (4)	0.0514 (3)	0.0512 (3)	−0.0002 (2)	0.0179 (2)	−0.00693 (19)
C1A	0.0543 (18)	0.0415 (16)	0.0383 (15)	−0.0025 (13)	0.0156 (13)	−0.0022 (12)
C9A	0.048 (2)	0.0385 (18)	0.0434 (17)	−0.0072 (15)	0.0143 (15)	−0.0046 (14)
C1B	0.063 (2)	0.0443 (17)	0.0482 (17)	−0.0116 (14)	0.0023 (15)	0.0032 (13)
C9B	0.0474 (19)	0.0451 (19)	0.0477 (18)	−0.0094 (15)	0.0093 (15)	−0.0068 (15)

### Geometric parameters (Å, º)

**Table:**

C2—C7	1.388 (2)	C12—C13	1.382 (2)
C2—C3	1.392 (3)	C12—H12	0.9300
C2—S1	1.7545 (19)	C13—C14	1.376 (2)
C3—C4	1.368 (3)	C13—H13	0.9300
C3—H3	0.9300	C14—O2	1.362 (2)
C4—C5	1.365 (3)	C14—C15	1.386 (2)
C4—H4	0.9300	C15—C16	1.367 (2)
C5—C6	1.360 (3)	C15—H15	0.9300
C5—H5	0.9300	C16—H16	0.9300
C6—C7	1.400 (3)	C17—O2	1.413 (3)
C6—H6	0.9300	C17—H17A	0.9600
C7—N1	1.415 (2)	C17—H17B	0.9600
C8—O1	1.222 (2)	C17—H17C	0.9600
C8—N1	1.363 (2)	N1—H1	0.8600
C8—C9B	1.505 (4)	S1—C1B	1.725 (3)
C8—C9A	1.516 (4)	S1—C1A	1.808 (3)
C10—C9A	1.262 (4)	C1A—C9A	1.484 (4)
C10—C9B	1.361 (4)	C1A—H1A	0.9700
C10—C11	1.464 (2)	C1A—H1B	0.9700
C10—H10A	0.9300	C1B—C9B	1.494 (4)
C10—H10B	0.9300	C1B—H1C	0.9700
C11—C12	1.395 (2)	C1B—H1D	0.9700
C11—C16	1.397 (2)		
			
C7—C2—C3	118.18 (18)	C12—C13—H13	120.1
C7—C2—S1	126.08 (14)	O2—C14—C13	124.28 (16)
C3—C2—S1	115.74 (14)	O2—C14—C15	116.08 (15)
C4—C3—C2	122.73 (19)	C13—C14—C15	119.64 (16)
C4—C3—H3	118.6	C16—C15—C14	119.87 (15)
C2—C3—H3	118.6	C16—C15—H15	120.1
C5—C4—C3	119.0 (2)	C14—C15—H15	120.1
C5—C4—H4	120.5	C15—C16—C11	122.34 (16)
C3—C4—H4	120.5	C15—C16—H16	118.8
C6—C5—C4	119.7 (2)	C11—C16—H16	118.8
C6—C5—H5	120.2	O2—C17—H17A	109.5
C4—C5—H5	120.2	O2—C17—H17B	109.5
C5—C6—C7	122.6 (2)	H17A—C17—H17B	109.5
C5—C6—H6	118.7	O2—C17—H17C	109.5
C7—C6—H6	118.7	H17A—C17—H17C	109.5
C2—C7—C6	117.89 (17)	H17B—C17—H17C	109.5
C2—C7—N1	128.43 (17)	C8—N1—C7	139.85 (15)
C6—C7—N1	113.67 (16)	C8—N1—H1	110.1
O1—C8—N1	117.68 (15)	C7—N1—H1	110.1
O1—C8—C9B	121.1 (2)	C14—O2—C17	117.57 (15)
N1—C8—C9B	119.10 (19)	C1B—S1—C2	98.80 (12)
O1—C8—C9A	117.03 (19)	C1B—S1—C1A	74.06 (15)
N1—C8—C9A	123.48 (18)	C2—S1—C1A	104.06 (12)
C9B—C8—C9A	27.63 (14)	C9A—C1A—S1	113.6 (2)
C9A—C10—C9B	31.63 (17)	C9A—C1A—H1A	108.8
C9A—C10—C11	132.4 (2)	S1—C1A—H1A	108.8
C9B—C10—C11	130.31 (19)	C9A—C1A—H1B	108.8
C9A—C10—H10A	113.8	S1—C1A—H1B	108.8
C9B—C10—H10A	104.7	H1A—C1A—H1B	107.7
C11—C10—H10A	113.8	C10—C9A—C1A	121.8 (3)
C9A—C10—H10B	102.4	C10—C9A—C8	118.6 (2)
C9B—C10—H10B	114.8	C1A—C9A—C8	119.6 (3)
C11—C10—H10B	114.9	C9B—C1B—S1	111.6 (2)
H10A—C10—H10B	38.2	C9B—C1B—H1C	109.3
C12—C11—C16	116.35 (16)	S1—C1B—H1C	109.3
C12—C11—C10	124.67 (15)	C9B—C1B—H1D	109.3
C16—C11—C10	118.94 (15)	S1—C1B—H1D	109.3
C13—C12—C11	121.95 (16)	H1C—C1B—H1D	108.0
C13—C12—H12	119.0	C10—C9B—C1B	127.4 (3)
C11—C12—H12	119.0	C10—C9B—C8	113.0 (2)
C14—C13—C12	119.80 (16)	C1B—C9B—C8	119.6 (3)
C14—C13—H13	120.1		
			
C7—C2—C3—C4	−0.6 (3)	C7—C2—S1—C1B	39.04 (18)
S1—C2—C3—C4	179.50 (17)	C3—C2—S1—C1B	−141.03 (17)
C2—C3—C4—C5	−0.1 (3)	C7—C2—S1—C1A	−36.62 (18)
C3—C4—C5—C6	0.5 (3)	C3—C2—S1—C1A	143.31 (16)
C4—C5—C6—C7	−0.1 (4)	C1B—S1—C1A—C9A	−17.3 (2)
C3—C2—C7—C6	0.9 (3)	C2—S1—C1A—C9A	78.0 (2)
S1—C2—C7—C6	−179.14 (14)	C9B—C10—C9A—C1A	−109.5 (6)
C3—C2—C7—N1	−178.87 (18)	C11—C10—C9A—C1A	−8.6 (6)
S1—C2—C7—N1	1.1 (3)	C9B—C10—C9A—C8	68.3 (4)
C5—C6—C7—C2	−0.6 (3)	C11—C10—C9A—C8	169.3 (2)
C5—C6—C7—N1	179.2 (2)	S1—C1A—C9A—C10	107.7 (4)
C9A—C10—C11—C12	−30.8 (4)	S1—C1A—C9A—C8	−70.1 (4)
C9B—C10—C11—C12	11.7 (4)	O1—C8—C9A—C10	34.1 (4)
C9A—C10—C11—C16	151.4 (3)	N1—C8—C9A—C10	−161.6 (3)
C9B—C10—C11—C16	−166.1 (3)	C9B—C8—C9A—C10	−71.9 (4)
C16—C11—C12—C13	1.9 (2)	O1—C8—C9A—C1A	−148.0 (3)
C10—C11—C12—C13	−175.93 (16)	N1—C8—C9A—C1A	16.3 (4)
C11—C12—C13—C14	−0.2 (3)	C9B—C8—C9A—C1A	105.9 (6)
C12—C13—C14—O2	177.88 (16)	C2—S1—C1B—C9B	−85.8 (2)
C12—C13—C14—C15	−1.9 (3)	C1A—S1—C1B—C9B	16.4 (2)
O2—C14—C15—C16	−177.57 (15)	C9A—C10—C9B—C1B	118.0 (6)
C13—C14—C15—C16	2.2 (3)	C11—C10—C9B—C1B	10.1 (6)
C14—C15—C16—C11	−0.4 (3)	C9A—C10—C9B—C8	−63.2 (4)
C12—C11—C16—C15	−1.6 (2)	C11—C10—C9B—C8	−171.13 (19)
C10—C11—C16—C15	176.40 (16)	S1—C1B—C9B—C10	−102.3 (4)
O1—C8—N1—C7	−179.9 (2)	S1—C1B—C9B—C8	79.0 (4)
C9B—C8—N1—C7	−16.1 (4)	O1—C8—C9B—C10	−32.2 (4)
C9A—C8—N1—C7	15.9 (4)	N1—C8—C9B—C10	164.6 (2)
C2—C7—N1—C8	−1.1 (4)	C9A—C8—C9B—C10	57.2 (4)
C6—C7—N1—C8	179.1 (2)	O1—C8—C9B—C1B	146.6 (3)
C13—C14—O2—C17	−7.0 (3)	N1—C8—C9B—C1B	−16.6 (4)
C15—C14—O2—C17	172.7 (2)	C9A—C8—C9B—C1B	−123.9 (6)

### Hydrogen-bond geometry (Å, º)

**Table:**

*D*—H···*A*	*D*—H	H···*A*	*D*···*A*	*D*—H···*A*
N1—H1···O1^i^	0.86	2.07	2.9291 (18)	177
C6—H6···O1^i^	0.93	2.45	3.263 (3)	146
C1*B*—H1*C*···O1^ii^	0.97	2.52	3.377 (4)	147

Symmetry codes: (i) −*x*, −*y*, −*z*; (ii) *x*, *y*+1, *z*.
