# Supplementary figures and images for: Crystal structure of (Z)-3-(4-meth­oxy­benzyl­idene)-2,3-di­hydro­benzo[b][1,4]thia­zepin-4(5H)-one
Source: Acta Crystallogr E Crystallogr Commun. 2015 Jan 1;71(Pt 1):o21–2. doi: 10.1107/S2056989014026267 (PMC4331881; doi:10.1107/S2056989014026267)

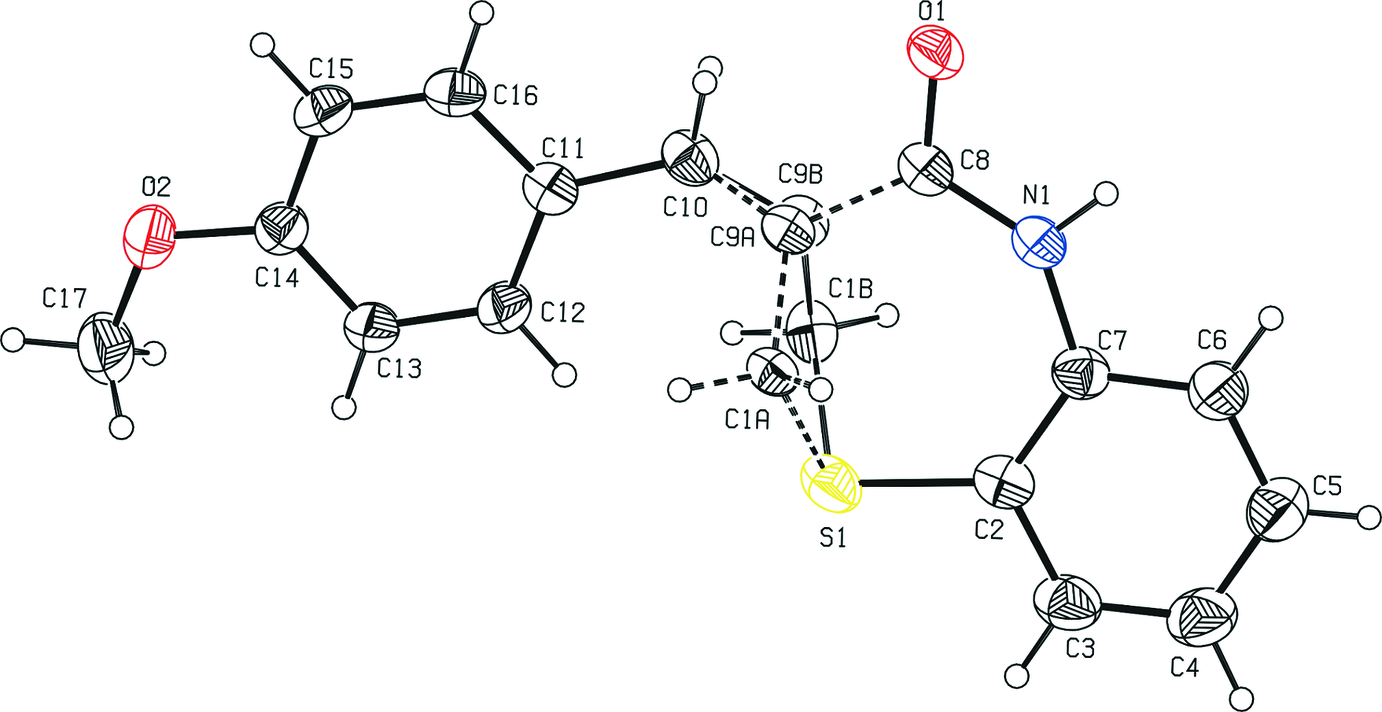

Supplement: Supplementary file 4 [file e-71-00o21-fig1.tif]

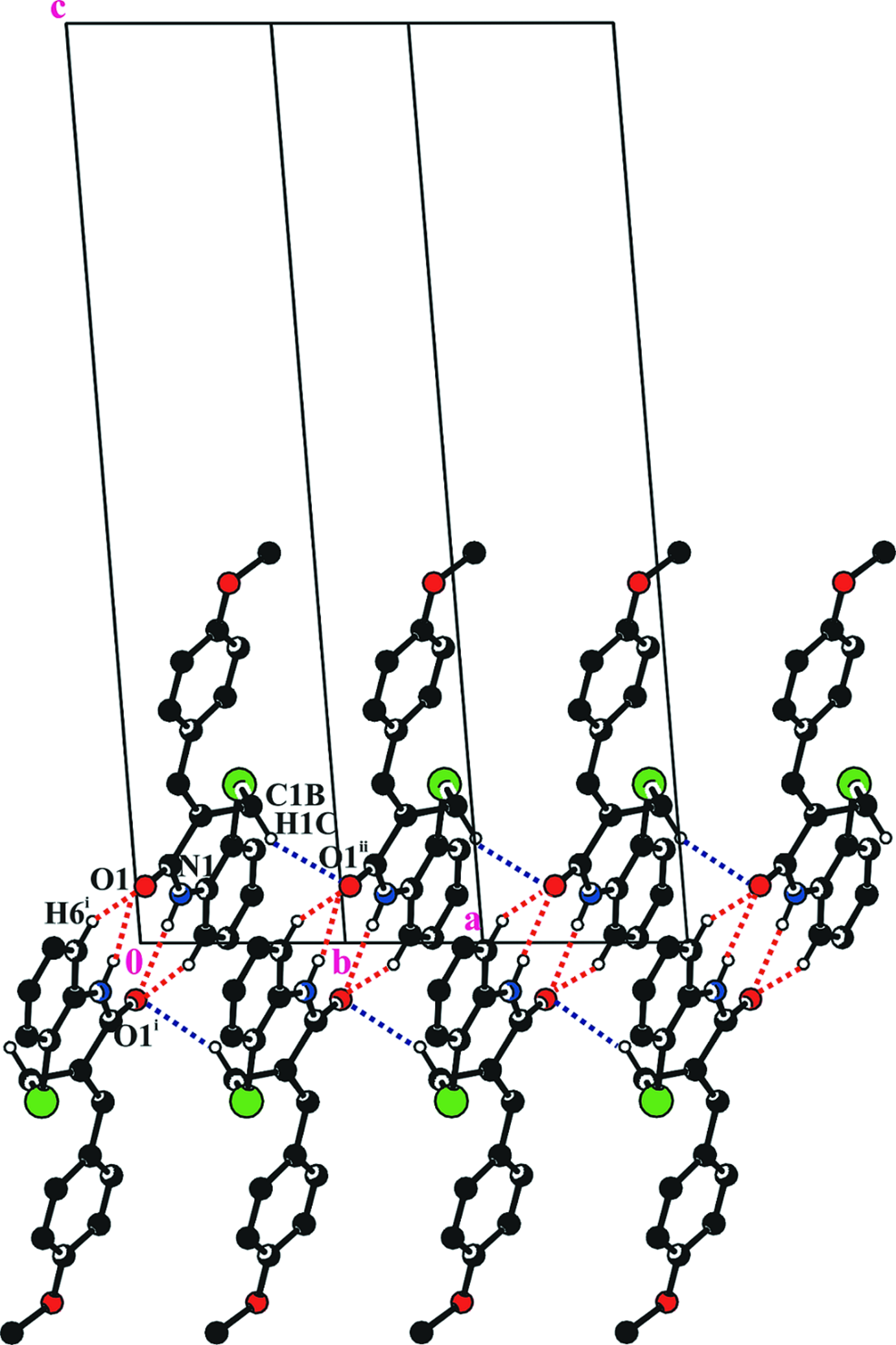

Supplement: Supplementary file 5 [file e-71-00o21-fig2.tif]
